# Neuronal morphology and synaptic input patterns of neurons in the intermediate nucleus of the lateral lemniscus of gerbils

**DOI:** 10.1038/s41598-023-41180-8

**Published:** 2023-08-30

**Authors:** Kathrin D. Wicke, Leon Oppe, Carla Geese, Anna K. Sternberg, Felix Felmy

**Affiliations:** Institute for Zoology, University of Veterinary Medicine Foundation, Hannover, Buenteweg 17, 30559 Hannover, Germany

**Keywords:** Auditory system, Brain

## Abstract

The lateral lemniscus encompasses processing stages for binaural hearing, suppressing spurious frequencies and frequency integration. Within the lemniscal fibres three nuclei can be identified, termed after their location as dorsal, intermediate and ventral nucleus of the lateral lemniscus (DNLL, INLL and VNLL). While the DNLL and VNLL have been functionally and anatomically characterized, less is known about INLL neurons. Here, we quantitatively describe the morphology, the cellular orientation and distribution of synaptic contact sites along dendrites in mature Mongolian gerbils. INLL neurons are largely non-inhibitory and morphologically heterogeneous with an overall perpendicular orientation regarding the lemniscal fibers. Dendritic ranges are heterogeneous and can extend beyond the nucleus border. INLL neurons receive VGluT1/2 containing glutamatergic and a mix of GABA- and glycinergic inputs distributed over the entire dendrite. Input counts suggest that numbers of excitatory exceed the inhibitory contact sites. Axonal projections indicate connectivity to ascending and descending auditory structures. Our data show that INLL neurons form a morphologically heterogeneous continuum and incoming auditory information is processed on thin dendrites of various length and biased to perpendicular orientation. Together with the different axonal projection patterns, this indicates that the INLL is a highly complex structure that might hold many unexplored auditory functions.

## Introduction

In Mammals, within the fiber bundle of the lateral lemniscus (LL), at least three major neuronal populations can anatomically be identified. These nuclei are termed according to their position dorsal (DNLL), intermediate (INLL) and ventral (VNLL) nucleus of the lateral lemniscus^1,2^. From these nuclei, the DNLL can be identified by its ventral location to the inferior colliculus (IC) and the fiber crossing of the LL with the commissure of Probst^2,3^. In gerbils, the DNLL appears roundish^3,4^. A thin layer of highly horizontally organized bipolar cells^3,5^ of unknown function defines the ventral boarder of the DNLL towards the INLL. Ventrally the INLL merges with the cell population of the VNLL. The VNLL stretches to the most ventral site of the LL and is composed of a globular and a heterogeneously shaped cell population^2,3,6^. While the anatomical border between the INLL and the VNLL appears less well defined, both populations differ in their transmitter content. Neurons in the VNLL are co-assigned to GABA and glycine transmitters and, in rodent and cat, the INLL to glutamate^7,8^, while in the bat INLL also glycinergic neurons were reported^9–11^. The transmitter content of INLL neurons in gerbils is so far undetermined.

The morphology of DNLL neurons was suggested to be multipolar and elongated^2,3,12,13^ with a large range of somatic sizes^12^. In the VNLL, multipolar or elongated neurons are distributed throughout the nucleus with dendrites ranging up to 400 µm^14^. In rodents, globular VNLL neurons are located at the ventro-lateral side^3,6,14^ and are presumably the targets of large endbulb synapses^6,15–18^. In the INLL, neurons were suggested to be multipolar or horizontally arranged^3^, but a detailed and quantitative description or single cell reconstructions have not been reported. Thus, it is unclear how they compare to the other neuronal populations in the lateral lemniscus.

In the VNLL, synaptic input structures are well-described for globular neurons receiving a large somatic, excitatory endbulb synapse^6,15–18^. Other synapses emerge from small and thick afferent fibers with different sized boutons^18,19^. For DNLL neurons, inputs arising from the contralateral side and lemniscal fibers form pericellular baskets^20^, suggesting that GABAergic synapses are localized over the entire cell surface, which is corroborated by identified GABAergic synapses at dendrites and soma^21^. At other locations in the LL the composition of synaptic structures and their location at distinct cellular compartments remains elusive. Again only in the DNLL and VNLL, the functional input patterns have been investigated. Globular VNLL neurons receive a large excitatory input^6,16,22,23^ while other VNLL neurons receive many small excitatory inputs^6^, and inhibitory currents are similar in size and kinetics throughout the VNLL^6^. In the DNLL, excitation is based on AMPA and NMDA receptor mediated currents^4,24–27^ and inhibition is largely GABA_A_ receptor mediated^28,29^. In the INLL, in vivo pharmacology indicated that inhibition is conveyed by ionotropic GABA and glycine receptors^30,31^, while excitatory inputs are unexplored. However, one function that was assigned to a subset of INLL neurons is in connecting auditory information across different sound frequencies^32^.

Here we assay the transmitter content, the single cell morphology and the distribution of synaptic inputs for neurons in the INLL in mature Mongolian gerbils. As in other rodents, INLL neurons appear largely non-inhibitory. Cells exhibit different morphologies and a wide range of dendritic sizes and shapes. INLL dendrites are overall perpendicular organized to the LL and in some cases extend beyond the medial border of the INLL. Excitatory input appears driven by VGluT1 and VGluT2 positive terminals. Inhibition appears dominated by GlyT2 contact sites but GABAergic terminals are also present. Excitatory and inhibitory contact sites show little clustering.

## Methods

### Animals

Mongolian gerbils (*Meriones unguiculatus*) of both sexes, based on Charles River hereditary background, were kept in the institute’s animal facility under 12 h light/dark cycle, fed ad libitum and were used between postnatal day (P) 28 and 40. Experiments were approved by the University animal welfare committee and animal welfare officer under the license number TiHo-T-2019–4 and TiHo-T-2021–4 and were compliant with German local and federal laws in accord with ARRIVE guidelines. We have used gerbils in this study, as these animals are a well-documented animal model for hearing research. The data presented in this study was obtained from a total of 15 gerbils. For single cell electroporation eight (four transversal cuts and four sagittal cuts), for single cell electroporation with post-hoc immunofluorescence labelling four animals and for standard immunofluorescence three animals were used.

### Single cell labelling

For single cell electroporation, acute slices containing the lateral lemniscus were prepared. Gerbils of P28-P45 were anesthetized with isoflurane and after decapitation, brains were rapidly removed in preparation solution. Preparation solution 1 for morphological reconstruction contained (in mM): 93 NMDG, 93 HCl, 30 NaHCO_3_, 1.2 NaH_2_PO_4_, 2.5 KCl, 25 glucose, 20 HEPES, 5 ascorbic acid, 3 myo-inositol, 3 sodium pyruvate, 10 MgCl_2_, 2 CaCl_2_ and was bubbled with O_2_/CO_2_ (95/5%) to achieve a pH of 7.4. Preparation solution 2 for electroporation with subsequent immunofluorescent labelling contained (in mM): 120 saccharose, 25 NaCl, 25 NaHCO_3_, 1.25 NaH_2_PO_4_, 2.5 KCl, 25 glucose, 0.4 ascorbic acid, 3 myo-inositol, 0.11 sodium pyruvate, 3 MgCl_2_, 0.1 CaCl_2_ and was bubbled with O_2_/CO_2_ (95/5%) to achieve a pH of 7.4. Brains were trimmed, fixed in the slice chamber and submerged with the respective preparation solution. Transversal or sagittal slices of 200 µm (for morphological reconstruction) or 400 µm (for contact site reconstruction) thickness that contained the lateral lemniscal nuclei were taken. Slices were incubated either for 30 min at 34°C in recording solution (after using preparation solution 1), or for 7 min at 36°C in recording solution (after using preparation solution 2). Extracellular recording solution contained (in mM): 125 NaCl, 25 NaHCO_3_, 1.25 NaH_2_PO_4_, 2.5 KCl, 25 glucose, 0.4 ascorbic acid, 3 myo-inositol, 0.11 sodium pyruvate, 1 MgCl_2_, 1.2 CaCl_2_ and was bubbled with O_2_/CO_2_ (95/5%) to achieve a pH of 7.4.

For single cell electroporation, slices were transferred to a set up with an upright BX50WI Olympus microscope equipped with a TILL Photonics Imaging system composed of an Imago CCD camera, a Polychorme IV and a control unit managed via TILLvisION software. Neurons were visualized with a 60 × or 40 × water immersion objective. Slices were continuously perfused with recording solution and kept at ~ 34°C. Patch-pipettes with a tip-diameter of about 1 µm were filled with 10 mM Alexa568 hydrazide or Alexa594 hydrazide dye (ThermoFisher scientific) dissolved in water. The dye filled pipette was pressed onto a visually selected neuron and a single ~ 15 ms long voltage pulse of ~ 15 V was applied. The trigger for the voltage pulse was delivered from a HEKA EPC10/2 amplifier to a stimulator unit (Model 2100, A-M systems). For re-slicing, cells were filled under whole cell configuration for at least 10 min using an intracellular solution containing (in mM): 145 K-gluconate, 15 HEPES, 4.5 KCl, 7.5 Na_2_-phosphocreatine, 2 Mg-ATP, 2 K_2_-ATP, 0.3 Na_2_-GTP and 0.5 K-EGTA, 0.3 Alexa568 hydrazide. Cell loading was visually verified. Slices with dye loaded cells were fixed in 4% paraformaldehyde (PFA) (for morphological reconstruction) or 3.7% PFA with 0.3% glutaraldehyde (for contact site reconstruction) over night. After fixation, slices were washed three times for 5 min in PBS. 200 µm slices were mounted with Vectashield and prepared for microscopy. 400 µm thick slices were mounted and readily used for overview microscopy. Images with a 2.5 × 0.075NA and a 20 × 0.5NA objective were taken to identify the position of the cells within the INLL and the shape of their soma and primary dendrites. These images were required for final re-assembly, because these 400 µm thick sections were re-sliced in 50 µm thick sections and prepared for immunofluorescence labelling.

### Immunofluorescence labelling

Gerbils were euthanized by inhalation of carbon dioxide and declared dead after 1–2 min of breathing arrest. Subsequently, they were transcardially perfused with cold Ringer solution containing heparin, followed by fixation with 3.7% PFA with 0.3% glutaraldehyde (for transmitter content labelling) or with 2% PFA containing 15% picric acid (for pre-synaptic labelling). Brains were removed, post-fixed overnight in PFA and washed with phosphate-buffered saline (PBS), adjusted to pH 7.4. Transversal slices of 50 μm were obtained with a Leica vibratome VT1200S. Sections from perfusions and from re-sliced electroporations were labelled in the same manner. Free-floating sections were washed in PBS, treated with sodium borohydride (1 mg/ml, for 10 min on ice) and subsequently with blocking solution (0.5% Triton, 1% bovine serum albumin and 0.1% saponin, diluted in PBS) for 30 min at room temperature. Sections were incubated for 2–3 days at 4°C in blocking solution containing primary antibodies (see Table 1). Sections were washed in 0.5% Triton and 0.1% saponin diluted in PBS and incubated in blocking solution containing secondary antibodies (see Table 2) for 4 h at room temperature. Sections were washed with PBS and mounted on microscope slides embedded in fluorescence mounting medium (VECTASHIELD©, Vector Laboratories).

**Table 1:** Primary antibodies used for histology.

Antigen	Host	Type	Used 2ndary AB	Dilution	Company	Cat#
VGluT1	Guinea Pig	Polyclonal	Alexa 488	1:2000	Synaptic Systems	135 304
VGluT1	Mouse	Monoclonal	Alexa 488	1:1000	Synaptic Systems	135 311
VGluT2	Guinea Pig	Polyclonal	Cy3	1:500 -1000	Synaptic Systems	135 404
GlyT2	Rabbit	Polyclonal	Alexa 488 AMCA	1:8000	Synaptic Systems	277 003
VIAT	Guinea Pig	Polyclonal	Cy3	1:8000	Synaptic Systems	131 308
MAP2	Chicken	Polyclonal	AMCA	1:1000	OriGene	TA336617
GABA	Mouse	Monoclonal	Alexa 488	1:200	Swant	mAB 3D5
Glycine	Rabbit	Polyclonal	Cy3	1:500	MoBiTec	1015GE

**Table 2:** Secondary antibodies used for histology.

Antigen	Conjugate	Host	Dilution	Company	Cat#
Anti-chicken	AMCA	Donkey	1:100	Dianova	703-156-155
Anti-mouse	Alexa488	Donkey	1:200—400	Dianova	715-545-150
Anti-rabbit	Cy3	Donkey	1:500	Dianova	711-165-152

### Image acquisition, alignment and three-dimensional reconstruction

The appropriate localization of the soma of electroporated neurons within the INLL was verified by epifluorescence overview microscopy. The LL fiber bundle was apparent by an increased autofluorescence excited with 488 nm. Images for single cell reconstruction were acquired with a confocal laser microscope (Leica TCS SP5, Leica Microsystems GmBH, Wetzlar, Germany) using a 40 × 1.25 NA oil immersion objective with an optical zoom of 2.5-fold. The magnification led to a pixel size of 0.303 × 0.303 µm. Z-stacks were taken with 0.29 µm step size generating a nearly cubic voxel. AMCA-fluorescence was excited with a 405 nm laser, Alexa488-fluorescence with an Argon Laser with the excitation tuned to 488, Alexa568-fluorescence with a HeNe laser tuned to 594 nm. Emission filters were adjusted to 410 – 470 nm for AMCA, 495 – 540 nm for Alexa488 and 598 – 700 nm for Alexa568. Images were acquired sequentially with a line averaging of 6 or 7. Images taken from standard free floating immunofluorescence labelling were taken with the same confocal laser microscope with 20 × 0.75 NA, 40 × 1.25 NA objective or for higher magnifications 63 × 1.4 NA objective.

Care was taken that the gain during image acquisition was adjusted so that all small cell compartments could be detected within the image stack. In this approach, the image stack that contains the soma and proximal dendrites lead to a largely over-illuminated soma. Therefore a second soma stack was taken where the gain was adjusted to be non-over-illuminating the soma. From these image stacks, the soma volume was reconstructed and used for further analysis. All other z-stacks that contained parts of a given cell were stitched together with the Fiji plugin^33^ to form an image of the entire optical reconstruction of a cell. Stitched image stacks containing labelled neurons were imported to Neurolucida360 (MBFBioscience) with the voxel size defined from the confocal reconstruction. In this image volume, neurons were manually reconstructed in three dimensions. As a central reference point the soma was digitalized first. From this starting point, individual processes were traced manually towards their distal end and the diameter was continuously adjusted. Nissl stains were imaged with a bright field microscope (Axioskop) with a 2.5 × 0.075 NA objective. Images were acquired with a USB camera (DFK 23UX249, Imaging Source) controlled by IC capture software. Images were post-hoc edited in Fiji and pairwise stitched^33^.

### Data analysis and statistics

Digitalized cells were transferred from NeuroLucida360 to Neurolucida Explorer for analysis. The neuron summary extracted the number of primary dendrites, number of nodes, total dendritic length, total dendritic volume and soma volume. A three-dimensional Sholl analysis with 10 µm concentric shell interval originating from soma center was carried out. We extracted the average diameter, cumulative length, cumulative area and number of intersections for each Sholl radii. For the wedge analysis in Neurolucida360, the reference angle was adjusted perpendicular to the lateral direction of the lateral lemniscus in image volumes and centered on the soma. The dendritic length within 30° wedges was extracted and summed in polar plots according to their orientation. Synaptic contact sites were visually detected by the overlay of dendritic fluorescence from cell labelling and VGluT1/2 or GlyT2 immunofluorescence. Here, we define a synapse as the sum of all contact sites between a single pre-synaptic axon with a post-synaptic partner. Thereby, a single contact site might be composed of multiple active zones. A contact site is therefore the close proximity, partial fluorescence overlap, of a single presynaptic marked junction with the post-synaptic element. For positive identification, at least two successive images of the z-stack were required to show this fluorescence overlay. Contact site location was determined in two ways. First, input location was determined as distance from soma center according to the Sholl distance of the concentric shells and the nearest neighbor tool of Neurolucida360 was used to extract the inter-contact sites distance. Second, the contact site location was mapped directly on the extent and shape of the dendrite. For this purpose, the number of pixels of the dendritic extent between soma and contact site location were measured and converted to µm. Each dendritic branch was mapped from the soma and contact sites on each branch were only counted once. The distance was expressed as relative distance of the length of the dendrite from soma to distal end.

Values obtained from Neurolucida360 and Neurolucida Explorer were exported to Excel or IgorPro and further processed. Statistical significance was assayed by t-tests. Data are shown as mean ± SEM and for inter-contact distance for the example neuron as medians. Average values for Sholl analysis was only derived from radii with a minimum of events from three cells. Correlations of morphological features were statistically analyzed in IgorPro with the linear correlation tool and their r and p values are given.

## Results

**Figure 1:**
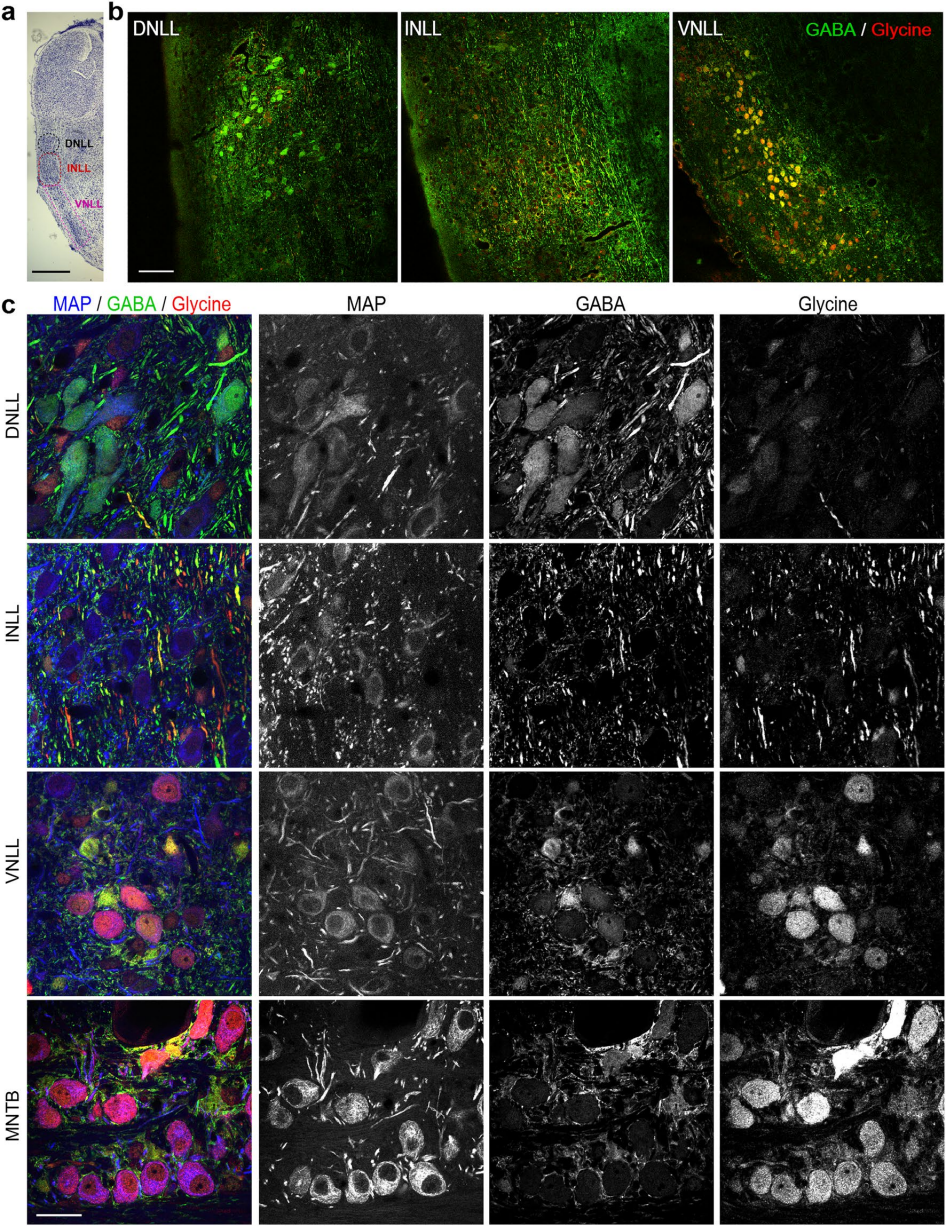
Position and transmitter content of nuclei in the lateral lemniscus. (**a**) Nissl staining of the lateral lemnisicus and inferior colliculus. Black circle indicates position of the DNLL, magenta circle the position of the INLL and pink square the position of the VNLL. Scale bar equals 1 mm. (**b**) GABA (green) and glycine (red) immunofluorescence in the DNLL, INLL and VNLL (from left to right). Scale bare equals 100 µm. (**c**) Co-labelling of MAP2 (blue), GABA (green) and glycine (red) immunofluorescence in the DNLL, INLL, VNLL and MNTB as indicated. Triple staining is given on the left, the single fluorescence of each channel is given in black in the indicated order: MAP-2, GABA and glycine. Scale bar equals 50 µm.

To anatomically address the INLL in Mongolian gerbils, Nissl staining on serial sections were carried out. The three LL nuclei could be identified (Fig. 1a) with the following landmarks. The DNLL resides ventrally of the IC with roundish appearance. The INLL follows ventrally to the DNLL and reaches to the position where the brain tapers to the ventral edge. The VNLL is slightly thinner compared to the other LL nuclei and stretches far ventrally where dark Nissl staining is present. These landmarks correspond to differences in transmitter content (Fig. 1b). DNLL neurons showed positive immunoreactivity to GABA but not glycine. INLL neurons mostly lack somatic immunoreactivity for both inhibitory transmitters. VNLL neurons appeared positive for both GABA and glycine immunoreactivity in individual cells, indicating the described co-release^34^. These findings are illustrated by higher magnification images (Fig. 1c). To confirm the specificity of antibody labelling, we added a labelling of the medial nucleus of the trapezoid body (MNTB), where MNTB neurons show their well-known glycinergic, non-GABAergic transmitter content. Thus, the profile of specific transmitter types in the gerbil LL matches that in cat^8^ and other rodents^7^.

**Figure 2:**
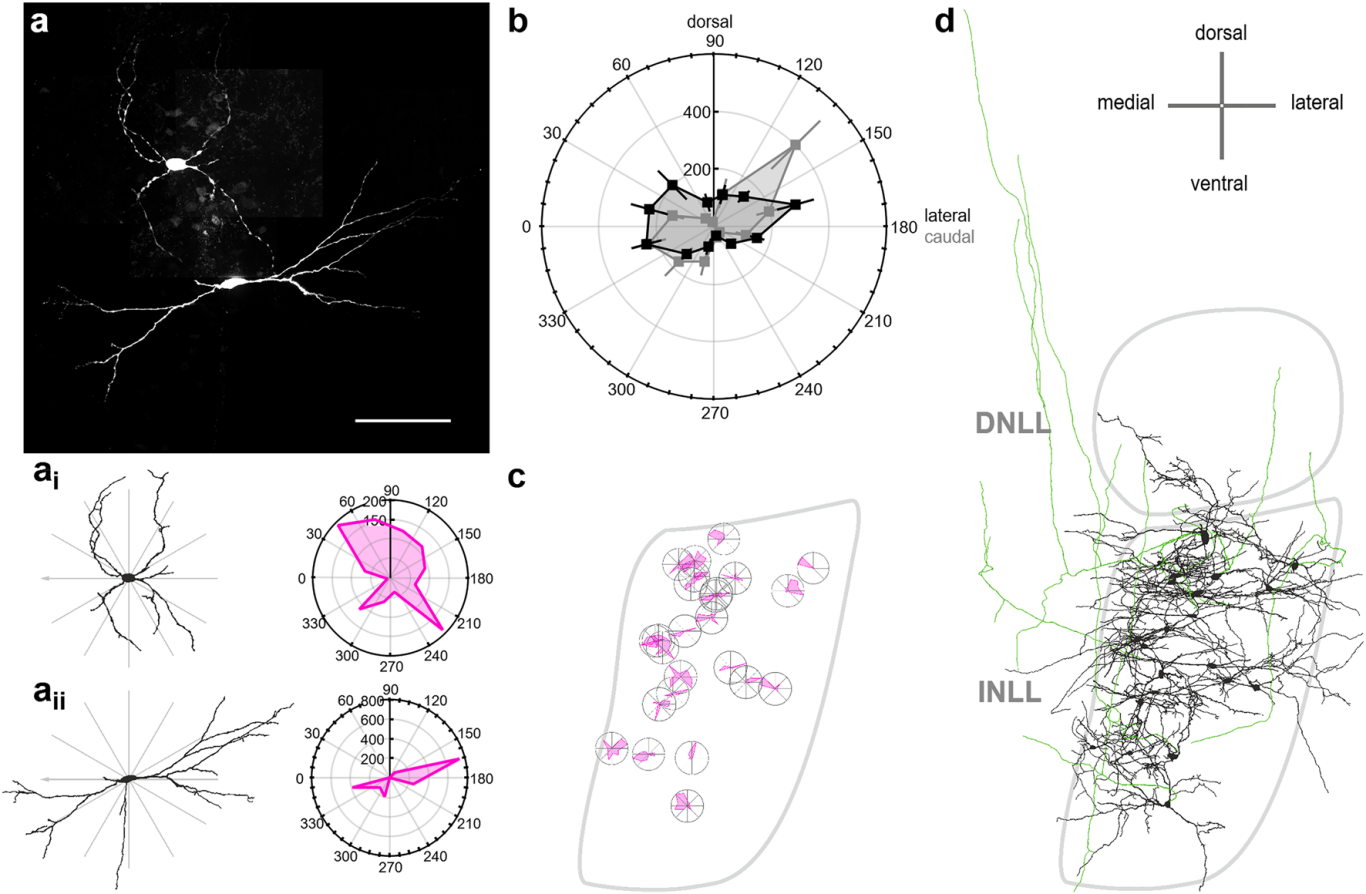
Neuronal orientation relative to the lemniscal fibers. (**a**) Examples of two electroporated INLL neurons. Scale bar equals 100 µm. (**a**_i_ and **a**_ii_) Left: Reconstructions from neurons shown in (**a**). Wedges used to extract dendritic length in a given orientation are indicated by gray lines. Right: Summed dendritic length of a given wedge presented as polar graph. Axis values are in µm. (**b**) Average dendritic length of all reconstructed INLL neurons (transversal: n = 25, black and sagittal: n = 17, gray) as a function of orientation. Axis values are in µm. (**c**) Polar plots of single cells at their position within a schematized INLL in the transversal plane. Sizes of polar plots are normalized to the maximal extension. (**d**) Overview of the position of reconstructed cells in the tranversal INLL. All labelled and reconstructed cells were added to the schematized INLL nucleus area (gray surrounding) and scaled to a similar proportion. Green compartments are axonal structures.

Neurons in the INLL were suggested to be horizontally oriented or multipolar^2,3^. To quantify the cellular extent, shape and orientation, we electroporated single neurons in acute brain slices and recovered their morphology (Fig. 2a). Images were acquired with the ventro-dorsal axis of the lateral lemniscus always being vertically oriented. The recovered neuron morphology was digitalized using Neurolucida 360 software (transversal: n = 25, sagittal: n = 17; Fig. 2a_i_, a_ii_). The orientation of the reconstructed cells was analyzed to gain insight into the dendritic arrangement within the lemniscal fiber tract. Towards this aim, the total dendritic length in a 30° wedge (Fig. 2a_i_, a_ii_) was cumulated. Some neurons were bipolar and horizontally orientated (Fig. 2a_ii_), while others were multipolar with little specific orientation (Fig. 2a_i_). Since we took care that all images were aligned with the ventro-dorsal axis of the lateral lemniscus, we were able to average the orientation of our sampled neurons. The overall dendritic orientation appeared horizontally orientated perpendicular to the fiber bundle irrespectively of the section plane (Fig. 2b). In case cells were recovered from transversal cuts, the average polar graph indicated that the lateral processes of INLL neurons are more focused in the perpendicular orientation to the ascending fibers than the medial extent (Fig. 2b). Thus, despite the heterogeneity of dendritic orientation, the overall population of INLL neurons appeared to have their dendrites orthogonally spread out in respect to the passing lemniscal fibers. No specific pattern of cell orientation was observed for cell locations within the transversal INLL template (Fig. 2c). Moreover, placing all reconstructed cells into a transversal template of the INLL nucleus (Fig. 2d), indicated that some neurons spanned the entire horizontal width of the nucleus and beyond.

**Figure 3:**
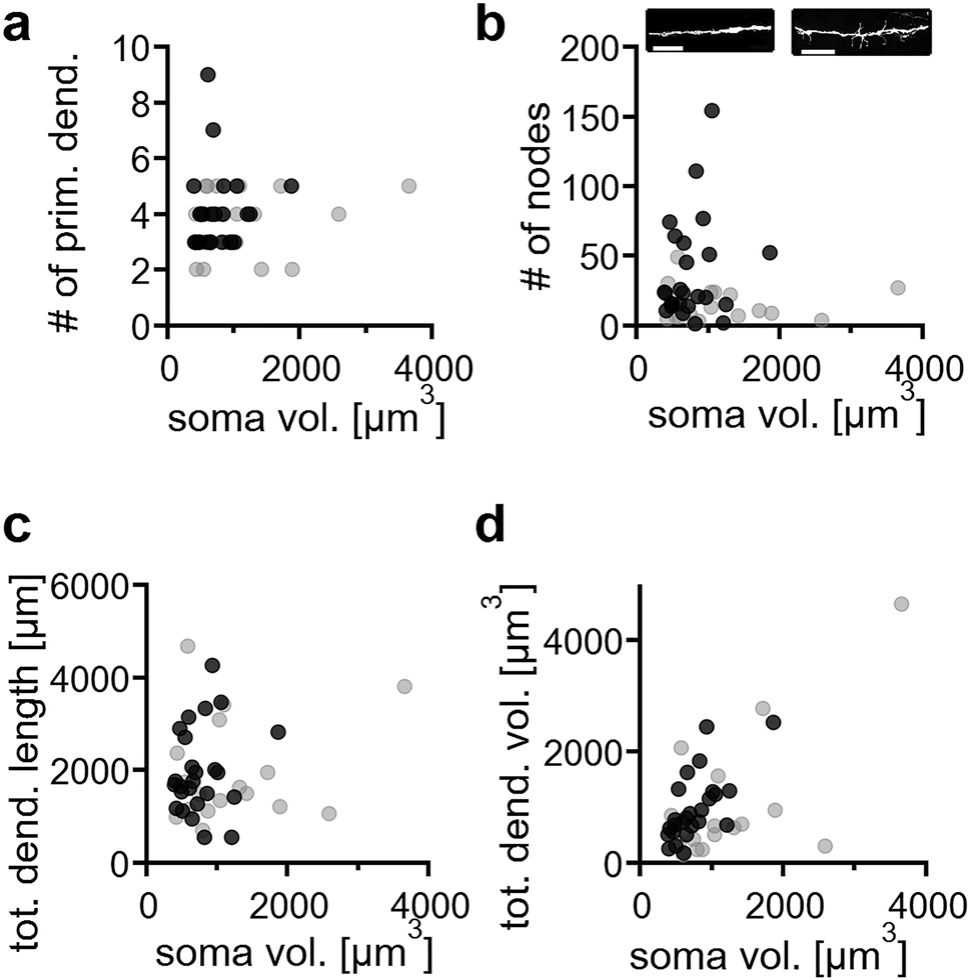
Basic morphological features of INLL neurons. (**a**) Number of primary dendrites emerging from a single soma as a function of the corresponding soma volume. Black symbols correspond to neurons recovered from transversal sections, gray symbols correspond to neurons recovered from sagittal sections. (**b**) Number of dendritic nodes as a function of soma volume. Insets show dendritic segments. Color code as in (**a**). One from a neuron without small dendritic protrusions and one from a neuron with such protrusions. Scale bars equals 10 µm. (**c**) Total dendritic length as a function of soma volume. Color code as in (**a**). (**d**) Total dendritic volume as a function of soma volume. Color code as in (**a**).

INLL neurons were further characterized by morphometric parameters extracted from the digitally reconstructed neurons (transversal: n = 25, sagittal: n = 17). INLL neurons had average somatic volumes of 766.3 ± 67.4 µm^3^ ranging from 396.7 to 1870.8 µm^3^ for transversal and 1232 ± 207 µm^3^ ranging from 424.0 to 3659.9 µm^3^ for sagittal sections. In average these neurons had 4.0 ± 0.3 and 3.8 ± 0.3 primary dendrites in transversal and sagittal slices respectively. The number of primary dendrites did not correlate with the soma volume (transversal: r = 0.094, p = 0.326; sagittal: r = 0.169, p = 0.259; Fig. 3a). The number of branch points, nodes, can approximate the elaboration of dendrites. INLL neurons showed a large heterogeneity in the node number (Fig. 3b). Some neurons had dendrites with little branching or side protrusions, while in some dendrites many small protrusions were observed (Fig. 3b). Since these protrusions were larger than 10 µm, they were not categorized as dendritic spines according to Hering and Sheng^35^. Neither the number of nodes (transversal: r = 0.201, p = 0.163, sagittal: r = 0.023, p = 0.465; Fig. 3b), nor the total dendritic length of INLL neurons correlated with soma volume (transversal: r = 0.174, p = 0.203, sagittal: r = 0.179, p = 0.245; Fig. 3c). However, the total dendritic volume correlated with the somatic volume (transversal: r = 0.674, p = 0.00011, sagittal: r = 0.646, p = 0.003; Fig. 3d), indicating that neurons with larger soma had overall thicker dendrites. These data of single cells did not indicate specific subpopulations, but rather appeared to form a continuum of morphological features. Therefore, no classification in specific neuronal morphologies was implemented.

**Figure 4:**
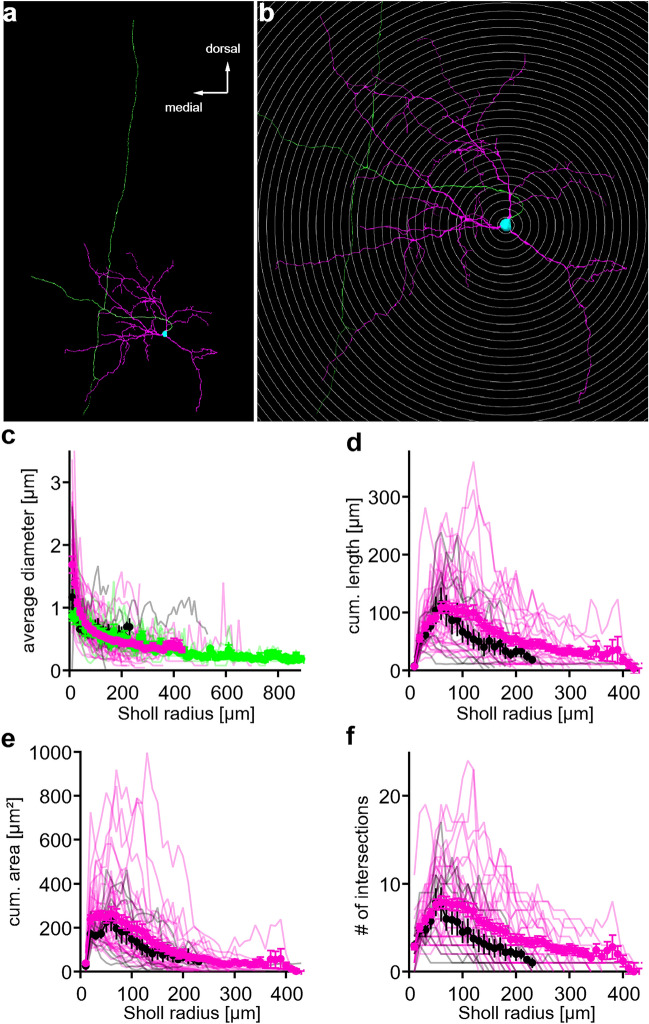
Sholl analysis of reconstructed INLL neurons. (**a**) Reconstructed example neuron. Dendrites colored in magenta, soma in cyan and axon in green. (**b**) Concentric shells of the Sholl analysis for the example neuron shown in (**a**). (**c**) Average diameter of neuronal processes within the Sholl radii. Dendritic data from whole-cell fillings colored in black (n = 8), dendritic data from single cell electroporation colored in magenta (n = 42), axonal data colored in green (n = 10). (**d**) Cumulative length of dendritic segments within the Sholl radii. Colors as in (**c**). (**e**) Cumulative area of the dendritic segment within the Sholl radii. Colors as in (**c**). (**f**) Number of intersections for each Sholl radius. Colors as in (**c**).

In 10 of our 42 single cell electroporated neurons (transversal: 7/25, sagittal: 3/17) an emerging axon was identified, by either an axonal bleb or its pathway to the DNLL or beyond. From these axons, 8 showed collaterals that not only indicated an ascending but also a descending information transfer, as exemplified in Fig. 4a and b. The reconstructed INLL neurons were further subjected to a Sholl analysis. Beside the electroporated neurons, where data obtained from transversal and sagittal sections were pooled, eight neurons were dye loaded under whole-cell conditions and their reconstructions were also used for Sholl analysis. Both approaches resulted in similar dendritic distributions along the Sholl radii. The average diameter of the dendrites dropped from the soma (electroporation: 1.68 ± 0.13 µm, n = 42; whole-cell filling: 1.17 ± 0.28 µm, n = 8; Fig. 4c) towards the distal dendritic end (electroporation 0.44 ± 0.05 µm, n = 33 and whole-cell filling: 0.58 ± 0.12 µm, n = 4 at 200 µm Sholl radius). Moreover, we found that dendrites in the INLL could extend up to 700 µm from the soma center. The dendritic diameter differed not from the extracted axonal diameter (electroporation dendrite 0.34 ± 0.04 µm, n = 11 and electroporation axon 0.45 ± 0.07µm, n = 8 at 350 µm Sholl radius; t-test, p = 0.36; Fig. 4c). Thus, the diameter of the different compartments did not allow their unambiguous categorization. The cumulated length and area of dendritic segments peaked (cumulated length electroporation: 109,4 ± 10.0 µm; whole-cell filling: 115.3 ± 27.8 µm; cumulated area: electroporation: 263.5 ± 30.9 µm^2^; whole-cell filling: 238.5 ± 54.3 µm^2^) at a Sholl radius of 60 – 70 µm (Fig. 4d and e). This peak indicated a local increase in arborisation and was similar to the stellate cells described in the VNLL^14^. The transient increase in arborisation was corroborated by the number of intersections within a Sholl radius (Fig. 4f). This value reached a maximal average of 7.9 ± 0.7 at 60 µm Sholl radius and 7.9 ± 1.6 at 50 µm for electroporated and whole-cell filled neurons respectively.

**Figure 5:**
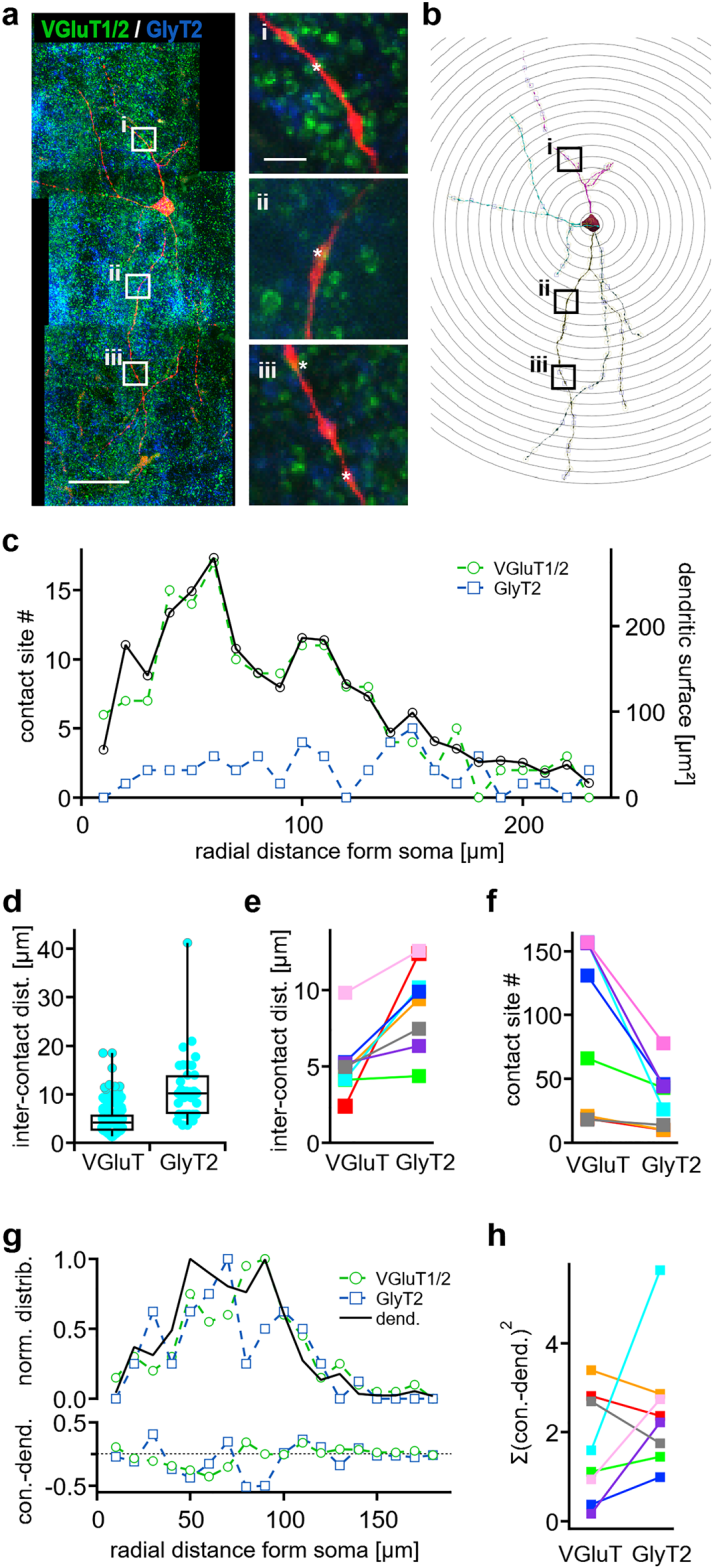
Distribution of synaptic input on dendrites of INLL neurons. (**a**) Single filled, reconstructed neuron (red) after re-slicing and labelling for VGlutT1/2 (green) and GlyT2 (blue). White squares indicate the position of the magnified insets. In the insets contact sites are marked with a white asterisk. Scale bars equal 50 µm and 5 µm for insets. (**b**) Digitalized neuron and the shells for the Sholl analysis for the neurons shown in (**a**). (**c**) Contact site number and dendritic surface within the Sholl radii. (**d**) Inter-contact distances for VGluT1/2 (VGluT) and GlyT2 labelled contact sites for the neuron shown in (**a**). Inter-contact distance is measured as the Euclidian distance between two contact sites on one dendrite. Inter-contact site distances across different dendrites were not taken into account. Box-plot shows median and quartiles. (**e**) Average inter-contact site distances. Each color corresponds to data from one neuron. (**f**) Number of VGluT1/2 and GlyT2 labelled contact sites along the sampled neurons (n = 8). Color code as in (**e**). (**g**) Top: Normalized contact site and dendritic surface distributions as a function of Sholl radius for the example neuron. Bottom: Difference between the normalized contact site (con.) and dendritic surface distributions as a function of Sholl radius. (**h**) Sum of the squared difference of contact site (con.) and dendritic surface distributions as shown in (**g**) bottom. Color code as in (**e**).

Our next aim was to quantify the distribution of synaptic contact sites along the dendrites of INLL neurons. From eight single neurons, we were able to determine the dendritic input pattern for VGluT1/2 and GlyT2 positive sites by immunofluorescence of resliced and realigned sections (Fig. 5a). Together with the Sholl analysis of these cells (Fig. 5b), the number of contact sites in each Sholl segment was counted. Thus, the number of inputs per dendritic surface area was obtained for VGluT1/2 and GlyT2 inputs for individual neurons (Fig. 5c). In the presented example (Fig. 5a to c), the number of glutamatergic inputs matched the distribution of the dendritic surface area closely. The glycinergic inputs were spread rather evenly over the dendritic extend. On this exemplary neuron, less GlyT2 than VGlut1/2-positive inputs were counted. In the next step, the distance between different VGluT1/2 and different GlyT2 contact siteswas extracted by the nearest neighbor tool of Neurlucida360 (Fig. 5d). For the example neuron, the excitatory distance was smaller (median 4.2 µm, 154 contact sites) compared to inhibition (median 10.2 µm, 37 contact sites, Fig. 5d). Similar to the exemplified cell, also the median inter-contact site distance for all analyzed cells was always smaller for VGluT1/2 (5.1 ± 0.4 µm) compared to GlyT2 (9.1 ± 1 µm) contact sites (Fig. 5e, t-test: p = 0.007). For all cells tested (n = 8), the number of VGluT1/2-positive sites (90.6 ± 23.1) was larger compared to the amount of GlyT2-positive (33.9 ± 5.9) sites (Fig. 5f, t-test: p = 0.038).

The difference in contact site numbers did not indicate whether the relative distribution profile of these inputs differ in respect to the dendritic surface distribution. Therefore, the normalized profiles along the radial distance from soma were calculated (Fig. 5g) and analyzed. To quantify possible differences of input distribution, the normalized VGluT1/2 and GlyT2 profiles were subtracted from the normalized dendritic surface distribution (Fig. 5g). Summing the squared values of these differences gave values for the deviance of input distribution from the dendritic surface distribution (Fig. 5h). There was no significant difference between the VGluT1/2 (1.64 ± 0.45) and GlyT2 (2.50 ± 0.54) distribution (p = 0.209, t-test), indicating that on average both input types follow the dendritic membrane distribution equally well.

**Figure 6:**
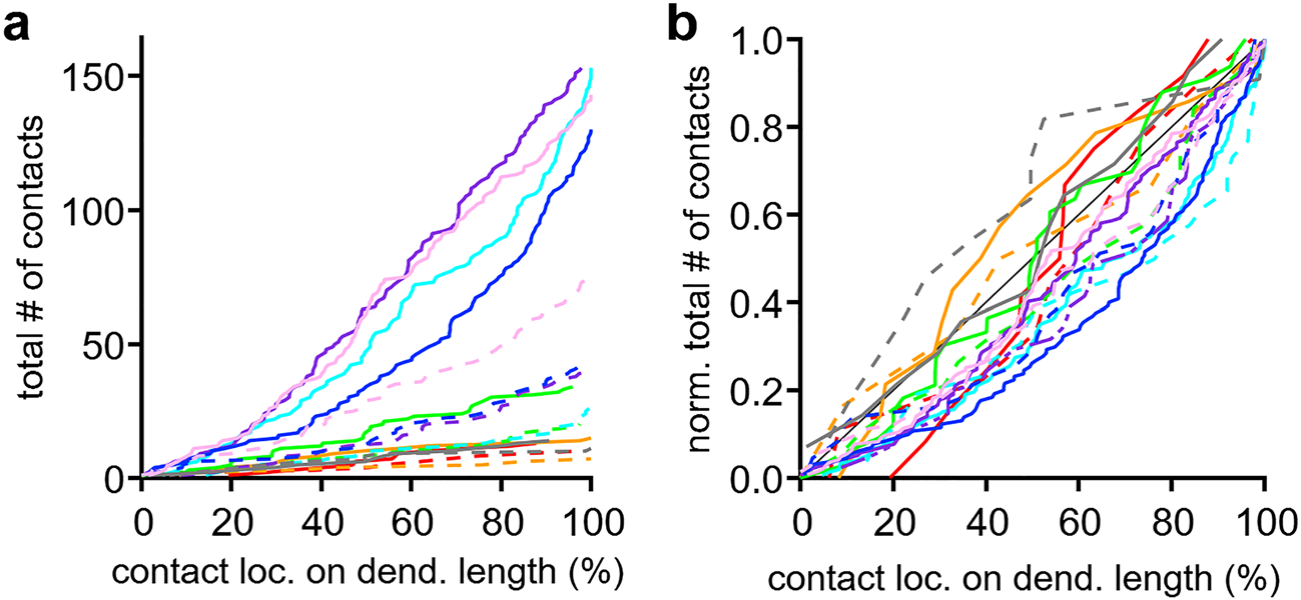
Synaptic location on dendrites of INLL neurons. (**a**) Total number of contact sites as a function of normalized dendritic location . Color code matches the data shown in Fig. 5. Solid lines correspond to VGluT1/2 and broken lines to GlyT2 positive contact sites. (**b**) Normalized number of contact sites as a function of normalized dendritic location. Color and line code as in (**a**). Black line indicates unity.

So far, the position and distance between contact sites was determined based on distances of Sholl radii but not on actual dendritic distances. Therefore, we analyzed the input location based on the actual dendritic morphology. We determined the relative position of each contact site along the dendrite. The dendritic length was normalized between soma and distal end, where 0% equals the beginning of the dendrite at the soma and 100% its end. Each branch ends therefore with 100% distance and thus, the dendritic arborisation of a single cell can be averaged. In Fig. 6b the number of VGluT1/2 and GlyT2-positive contact sites is culminated over their position on the normalized dendritic extent. A steep increase indicates a higher local density of synaptic inputs. Mostly we observed a steady increase in the number of VGluT1/2 and GlyT2-positive contact sites suggesting a homogeneous distribution along the dendrite. To corroborate this notion, the normalized number of VGluT1/2 and GlyT2-positive contact sites was mapped to their distance along the normalized dendritic tree (Fig. 6b). Both, inhibitory and excitatory inputs were, apart from one case, present at the soma and ended within the last 15% of dendritic length. Thus, both input types are distributed over the entire dendrite. However, for most cells the steepness of the distribution is lower in about the first 25% of the dendrite compared to distances between 70 and 90% , indicating that the synaptic density was lower at proximal than on distal dendrites.

**Figure 7:**
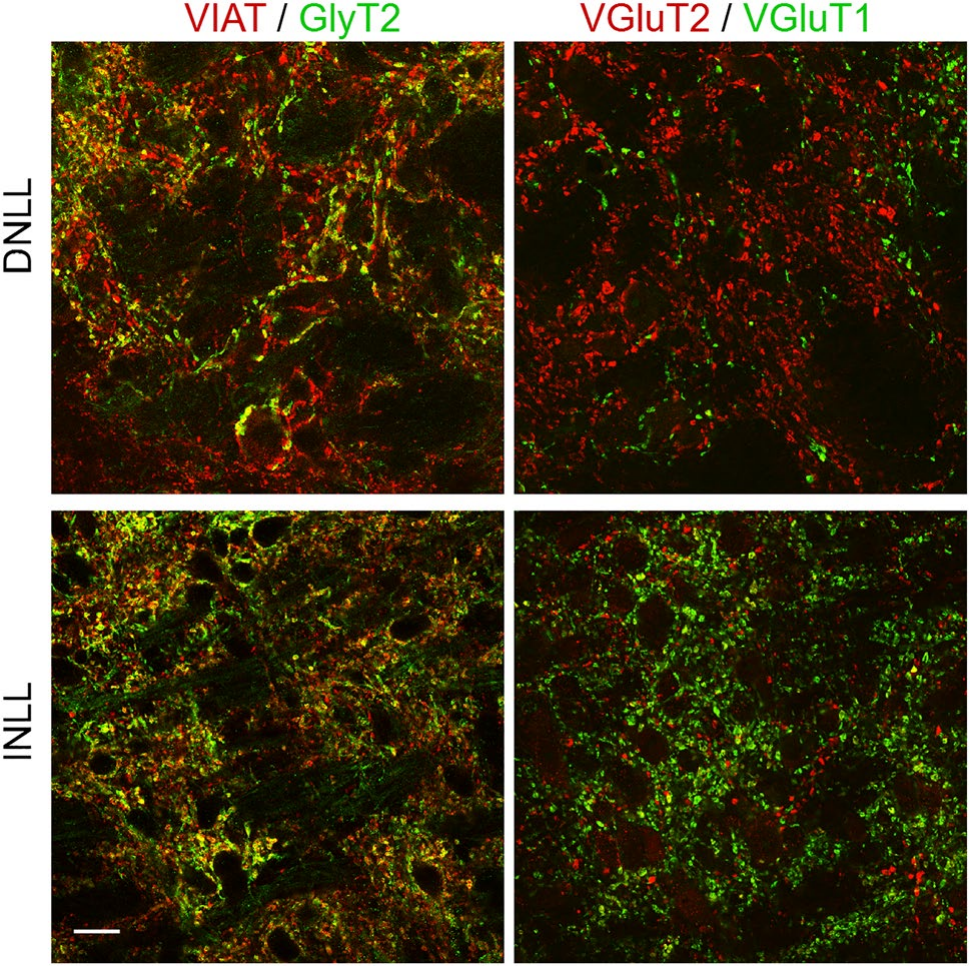
Composition of excitatory and inhibitory contact sites in the INLL. Left: Co-labelling of VIAT (red) and GlyT2 (green) in the DNLL (top) and the INLL (bottom). Right: Co-labelling of VGluT2 (red) and VGlut1 (green) in the DNLL (top) and the INLL (bottom). Scale bar equals 20 µm.

Finally, we seek to identify the molecular composition of the synaptic input types. Therefore, we immunofluorescently labelled sections for VGluT1 together with VGluT2 and VIAT together with GlyT2 to distinguish specific excitatory and inhibitory contact sites respectively. In sections containing INLL and DNLL the inhibitory inputs distributed differently (Fig. 7). As expected from the DNLL’s prominent reciprocal GABAergic connectivity^1,36,37^, VIAT was more present than GlyT2 in the DNLL. In the INLL, GlyT2 appeared the dominant marker for inhibitory contact sites (Fig. 7). This corroborated the different input nuclei of the INLL and DNLL^1,36^. The different input patterns in the DNLL and INLL were also present for glutamatergic inputs. VGluT1 prevails in the INLL and VGluT2 in the DNLL (Fig. 7). Thus, excitation appeared to arise from different sources in DNLL and INLL. Moreover, our estimated contact site distributions (Fig. 5 and 6) were based on the major synaptic input marker in the INLL.

## Discussion

Here we characterize the anatomy of INLL neurons in Mongolian gerbils. We provide landmarks for the INLL in gerbils, confirming that these neurons were mainly non-inhibitory, and therefore classified as excitatory. Inhibitory inputs to INLL neurons were more glycinergic and less GABAergic compared to the DNLL. Both inhibitory and excitatory inputs to INLL neurons distribute along the dendrite with overall less inhibition compared to excitation. Neurons of the INLL come in manifold morphologies and the dendritic orientation of the overall population appears orthogonal to the lateral lemniscus.

In gerbils, the INLL can be addressed as the cell population ventral to the DNLL and H-cells^3,5^ stretching ventrally to the position where in transversal sections the edge of the brain begins to taper towards its ventral side. In this region, the neurons appear neither GABA- nor glycinergic in gerbils. This lack of inhibitory transmitter phenotype is consistent with the description in rodents^7^ and cat^8^, but contrasts bats, where at least a fraction of INLL neurons are marked for inhibitory transmitters^9–11^. Thus, we conclude that INLL neurons in gerbils are largely glutamatergic with possibly a fraction of dopaminergic neurons that recently have been described in this lemniscal area in rats^38^. INLL neurons receive glutamatergic inputs that exhibit predominantly VGluT1-positive contact sites. Thus, the origin of ascending excitation originates mainly from the cochlear nucleus and not the SOC, because medial and lateral superior olivary neurons are positive for VGluT2 expression^7^. This molecular pattern is consistent with the physiological data that present INLL neurons as largely monaural^39,40^. The presence of both GABA- and glycinergic contact sites in the INLL indicates that both transmitters play a role in sound processing. This interpretation agrees with in vivo pharmacology in bats that indicated both GABA- and glycinergic effects on sound evoked discharges in the INLL^30,31^. Moreover, the mixed presence of GABA- and glycinergic contact sites in the INLL contrasts the dominant GABAergic inputs in the DNLL.

INLL neurons were described as multipolar and horizontal^3^ or fusiform cells^2^. Our reconstructions does not indicate a clear cut off between bipolar or multipolar neurons or other morphological features. Rather it appears that there is a continuum of morphological variations. Since we relate the different morphologies to a continuum rather than to specific appearances, we do not introduce a categorization of different cell types, as has been presented for other LL nuclei^13,14,20^. Importantly, no clustering of specific neuronal morphologies occurs within the nucleus and cells of different shapes distributed throughout the INLL. In various cases, the dendrites of the reconstructed neurons progressed beyond the apparent medial or dorsal border of the INLL. This might imply that neurons in the INLL receive non-auditory and possibly auditory inputs not only within the lemnsical fiber bundle and therefore also from sources outside the auditory lemniscus pathways.

Somatic volumes of INLL neurons show a wide heterogeneity covering a whole magnitude, and therefore show a similar heterogeneity compared to DNLL neurons in cat^12^. The dendrites of rat VNLL neurons have been quantified by a Sholl analysis before^14^. A similar value of maximal Sholl radius between these VNLL neurons and our INLL neurons exists. Moreover, the number of intersections is similar between our INLL and these VNLL neurons, with the exception of more dendritic branches in bushy cells of the VNLL^14^. Thus, the neuronal size and morphology seems similar between the non-bushy cells of the VNLL, with neurons from the INLL. Whether these neurons have also biophysical or functional similarities remains to be elucidated. This similarity of cell morphology between VNLL and INLL neurons adds to the difficulty to delineate a border between both nuclei and requires the use of other landmarks.

Our analysis shows that the sum of INLL dendrites orientats perpendicular to the fibers of the LL. Elongated neurons are mostly horizontally oriented, but also multipolar neurons emit their dendrites sometimes more in this orientation. The perpendicular orientation of dendrites to the lemniscal fiber bundle has been noted also in bats^2^ and is apparent in Golgi stains from gerbils^3^. In gerbils, the ascending lemniscal fiber bundle appears only partially tonotopically organized with lower frequencies located at the lateral and higher frequencies at more central positions^41^. Dendrites oriented perpendicularly across the entire INLL will be able to contact passing fibers carrier different frequency information. Therefore, it might be speculated that perpendicular oriented dendrites form synapses beyond the classical tonotopical arrangement and grab information across different frequency bands. Thus, the output of INLL neurons might not be narrowly tuned to a specific frequency. Indeed morphological evidence is provided from bats, where INLL neurons appeared less restricted to tonotopical projection patterns^2^. Moreover, the tontopical tuning of INLL neurons is less clearly conserved compared to nuclei of the superior olivary complex^42^, or the DNLL^40,43–46^. Again, in bats, the tonotopy of the INLL appears patchy^17,39,47^. In rodents, the tonotopy might be ventro-dorsally organized, however only a limited amount of in vivo data are present^40^ and do not fully agree with anatomical data^41^. In cat, again partially contradicting data about the tonotopy from in vivo recordings^48^ and anatomical studies are present^49^. The perpendicular orientation of INLL dendrites however might play a role in the functionality of INLL neurons and might underlie the weak tonotopical tuning. This speculation about a weak tonotopical arrangement of the INLL is consistent with reports of cross-frequency integration in INLL neurons^32^ and broad frequency tuning^31^. These functional features might be supported by information transfer across different frequencies from ascending frequency bands. Thus, a perpendicular orientation of dendrites to passing fibers might facilitate input detection across different ascending frequency areas and hence cross-frequency integration.

In INLL neurons, the synaptic density was larger for excitation compared to inhibition, judged by VGluT1/2 and GlyT2-positive co-localization with the dendrite or soma. However, it should be considered that each contact site might encompass more than a single active zone. Furthermore, GlyT2 will not cover all inhibitory inputs because of the presence of GABAergic synapses that are detected as VIAT/GlyT2-negative contact sites. Therefore, the absolute inhibitory density will be larger. Whether the total amount of inhibition is larger compared to the overall excitation remains unresolved. Because we detected more GlyT2 than VIAT/GlyT2-negative contact sites, we assume that GABAergic inputs are less pronounced.

Both, the density of excitation and GlyT2 mediated inhibition follow the size of the dendritic surface area. However, it appears that at the beginning of the dendrite GlyT2 positive contact sites are slightly more present compared to excitation, while at the far distal end of dendrites both excitation and inhibition are present in the same manner. A well-known example in the auditory pathways for somatic inhibition are the neurons of the medial superior olive (MSO). In MSO neurons, inhibition is more or less restricted to the soma^50–52^ and the restriction to the soma is species-dependent^52^, while excitation is distributed along the bipolar dendrites and soma^50,51,53^. This sub-cellular segregation is regarded to be functionally relevant for ultra-fast coincidence detection and binaural hearing^54^. Since in INLL neurons the somatic bias for inhibition is small, inhibition might serve other functions for postsynaptic integration compared to MSO neurons, irrespectively of neuronal morphology. In the auditory brainstem, synaptic distributions have been studied also in fusiform neurons of the dorsal cochlear nucleus^55^. In these cells the density of inputs increases with distance and larger inputs were observed at more basal dendrites and somata. The resolution of our images does not allow to estimate differences in synaptic size along the dendrite. Therefore, no direct comparison to fusiform cells can be drawn.

The distribution of contact sites along the dendritic extent highlights that sound processing in these neurons is based on dendritic integration. Since we do not observe a different distribution between distal or proximal dendritic branches, inhibitory and excitatory inputs are likely to be integrated in individual dendritic compartments. Therefore, dependent on the input pattern, dendritic integration might activate a full dendrite or only sub-regions that locally process input strength. The slight somatic bias of inhibition might serve as the standard shunt for suppressing dendritic evoked excitation. It remains to be seen, whether the large morphological heterogeneity of INLL neurons follows a specific structure–function relationship as proposed for VNLL neurons^14^ or similar to DNLL neurons where no structure–function relationship is indicated^13^.

In a small fraction of neurons, we could unambiguously identify axonal compartments. These axons projected dorsally and ventrally and in some cases appeared to have small local collaterals. Our experimental approach does not allow to trace projection axons to their final destination. Nevertheless, the initial projection patterns indicated that INLL neurons send their output to ascending structures, such as the DNLL and inferior colliculus. These ascending structures have been identified as targets of the INLL^1,2,36^. Our single cell labelling also identifies potential descending axonal compartments. Thus, INLL neurons are likely to be connected to descending structures of the superior olivary complex or cochlear nucleus. Since INLL neurons receive inputs from these structures^1,36^ and the inferior colliculus, it suggests that INLL neurons might form a central hub for looping sound information with other auditory nuclei. Together with the local axonal branching, this indicates that the INLL is a highly connected nucleus regarding its input and output targets. Such complex input–output structures suggest that INLL neurons are a center for computing complex monaural signals.

## Data Availability

The datasets generated during and/or analyzed during the current study are available from the corresponding author on reasonable request.
